# Concurrent Anterior Cruciate Ligament and Medial Patellofemoral Ligament Reconstruction: A Case Report and Literature Review

**DOI:** 10.7759/cureus.7717

**Published:** 2020-04-18

**Authors:** Vijay Shankar, Syed Natiq Hussain, Santosh Sahanand, David Rajan

**Affiliations:** 1 Orthopaedics, Golden Jubilee National Hospital, Glasgow, GBR; 2 Orthopaedics, Ortho One Orthopaedic Speciality Centre, Coimbatore, IND

**Keywords:** anterior cruciate ligament (acl), medial patellofemoral ligament, arthroscopy, hamstring autograft

## Abstract

Anterior cruciate ligament (ACL) insufficiency in combination with patellar instability are rare occurrences and are difficult to treat. Failure to address patellar instability in such cases may place excessive strain on ACL graft leading to graft rupture. We present three such cases treated by concurrent ACL and medial patellofemoral ligament (MPFL) reconstruction with hamstring tendon autografts. Two patients had MRI evidence of MPFL injury and one patient had intact MPFL on MRI. All patients had good outcome without any residual instability at final follow-up.

## Introduction

Anterior cruciate ligament (ACL) is the primary restraint to anterior translation of tibia and is one of the most commonly injured ligaments. It usually does not occur in isolation and takes place in conjunction with meniscal (50%) and collateral ligament (20%) injuries [1]. These concomitant injuries are usually addressed during ACL reconstruction and have good outcomes [2]. Medial patellofemoral ligament (MPFL) is the primary restraint to lateral patellar translation at 0-30 degrees flexion [3]. Management can be in the form of repair, reconstruction, or bony procedures [4]. The combination of ACL and MPFL tear is rare with only a few cases reported in literature [5-7]. The outcomes of simultaneous ACL reconstruction and patellar stabilization are unclear due to limited data available on these injuries [5-6]. We describe the surgical technique and follow-up of three such cases of this combined injury pattern managed with concurrent ACL and MPFL reconstruction using hamstring tendon autografts.

## Case presentation

Case 1

A 35-year-old female complained of pain and instability in right knee following twisting injury a year back. Clinical examination revealed positive Lachman, grade I pivot shift, and grade II anterior drawer. Her knee range of motion (ROM) was near full with a positive patellar apprehension on the affected side. Beighton score was normal.

MRI evidence showed complete ACL tear and MPFL tear from femoral attachment. She underwent arthroscopic ACL along with open MPFL reconstruction.

Case 2

A 39-year-old female presented to outpatient department with pain and instability in right knee following slip and fall two weeks back. Mechanism of injury was of valgus and external rotation. She had a childhood history of bilateral patellar dislocation. Clinical examination revealed positive Lachman, grade II pivot shift, and grade II anterior drawer. Her knee ROM was full with a positive patellar apprehension on the affected side. Beighton score was normal.

Plain radiographs were normal. MRI evidence showed complete ACL tear and MPFL tear from femoral attachment. CT scans of both lower limbs revealed normal bony alignment and tibial tuberosity trochlear groove (TTTG) distance. We went ahead with arthroscopic ACL along with MPFL reconstruction.

Case 3

A 28-year-old female came with complaints of left knee pain and instability following valgus injury while dancing three weeks back. Since then she had experienced four episodes of patellar dislocation. Clinical examination revealed positive Lachman, grade II pivot shift, and grade III anterior drawer. She had a positive patellar apprehension test with terminal restriction of knee ROM. We arrived at a clinical diagnosis of ACL and MPFL insufficiency. Beighton score was normal.

**Figure 1:**
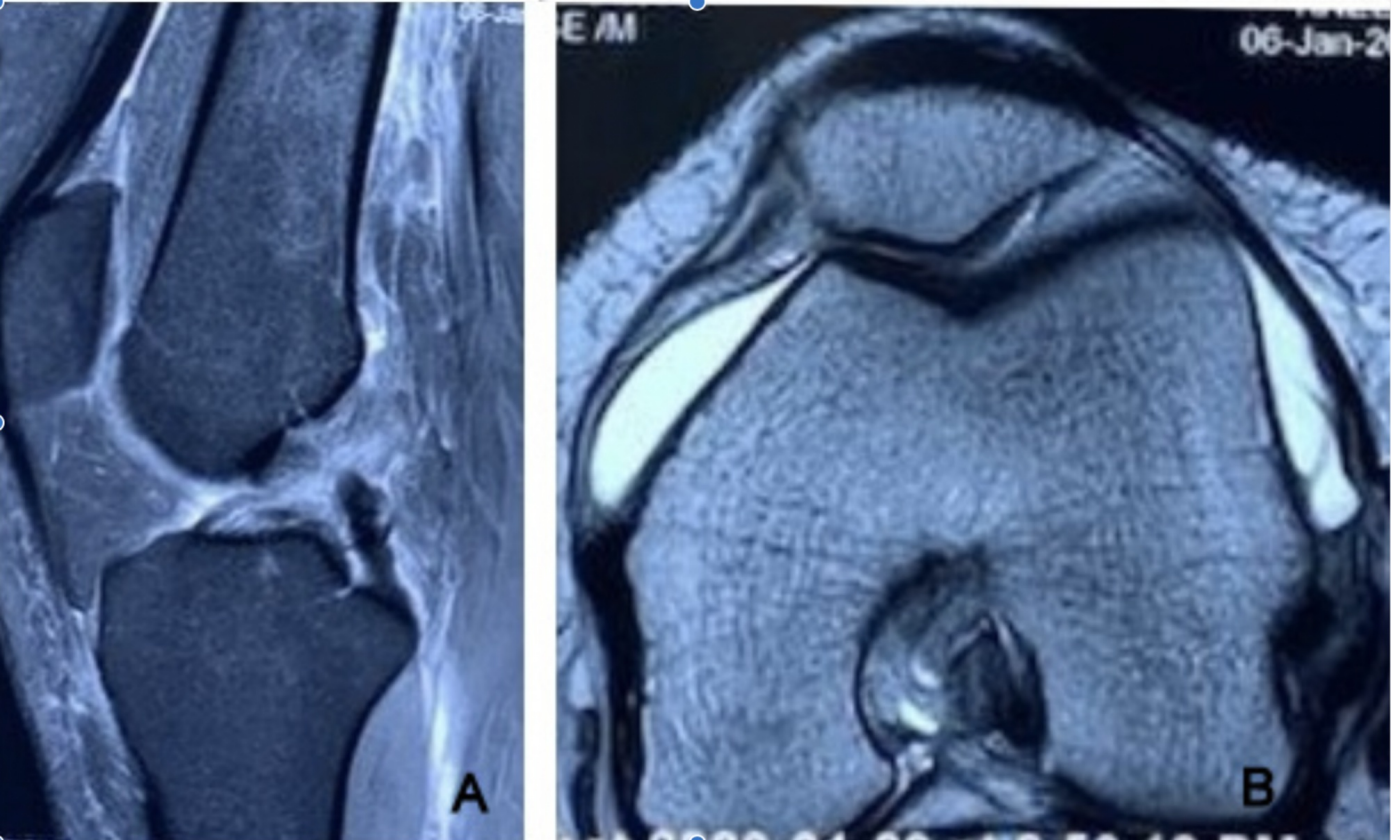
MRI of knee joint: A) sagittal view showing complete ACL tear and B) axial view showing intact MPFL. ACL, anterior cruciate ligament; MPFL, medial patellofemoral ligament

**Figure 2:**
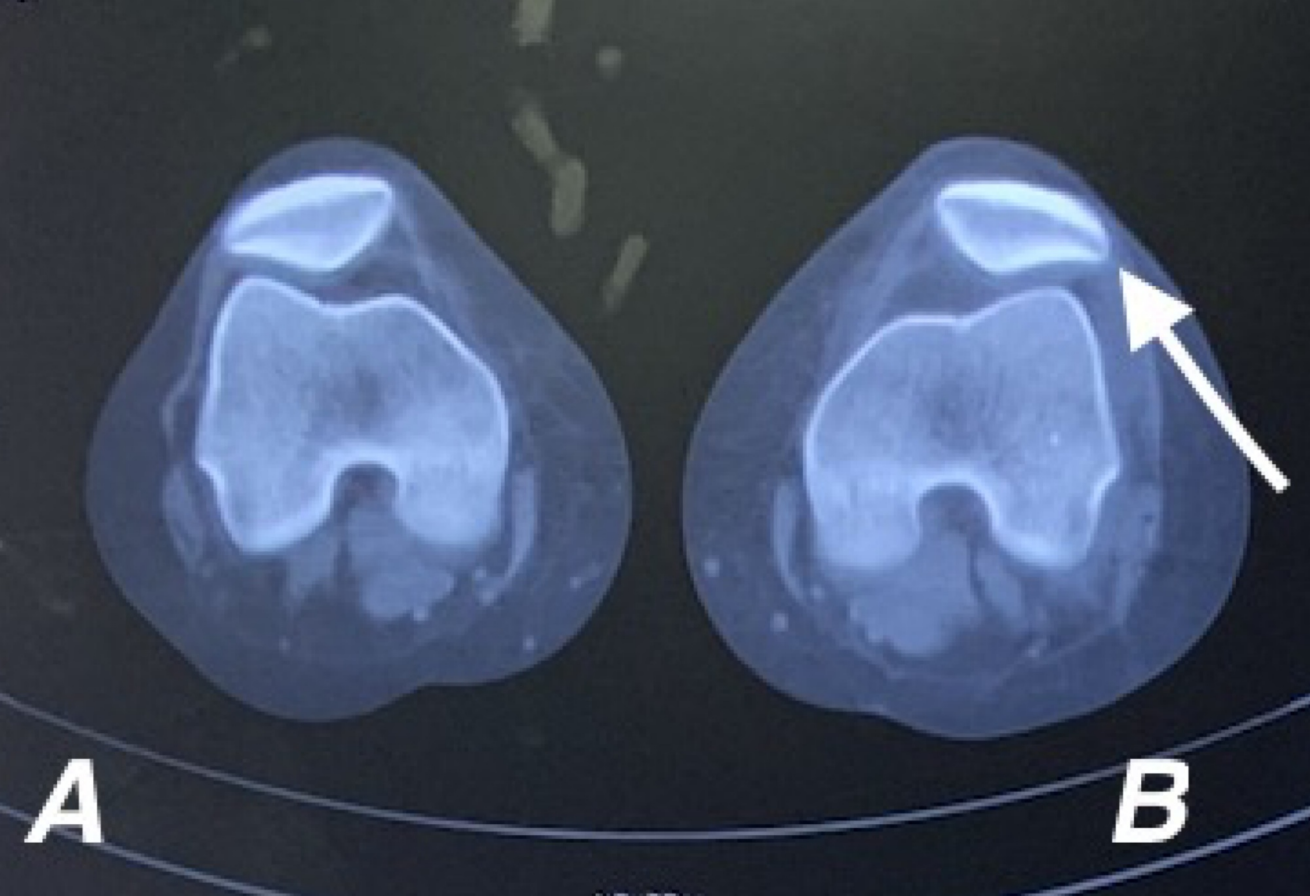
Axial CT cuts of A) right and B) left knees showing left lateral patellar tracking.

Plain radiographs were normal. MRI evidence revealed complete ACL tear from femoral attachment (Figure 1A). However, MPFL was normal and no classical contusion pattern was also seen (Figure 1B). CT scans showed normal TTTG and bony alignment with patellar maltracking (Figure 2). In view of recent episodes of recurrent patellar instability, positive patellar apprehension, and patellar maltracking we went ahead with MPFL reconstruction along with ACL reconstruction.

Surgical technique

Under spinal anesthesia, examination under anesthesia (EUA) was done to confirm positive Lachman, pivot shift, and lateral patellar translation. The patient was positioned supine on operating table with lateral thigh and foot post. Pneumatic tourniquet was applied to both the thighs.

**Figure 3:**
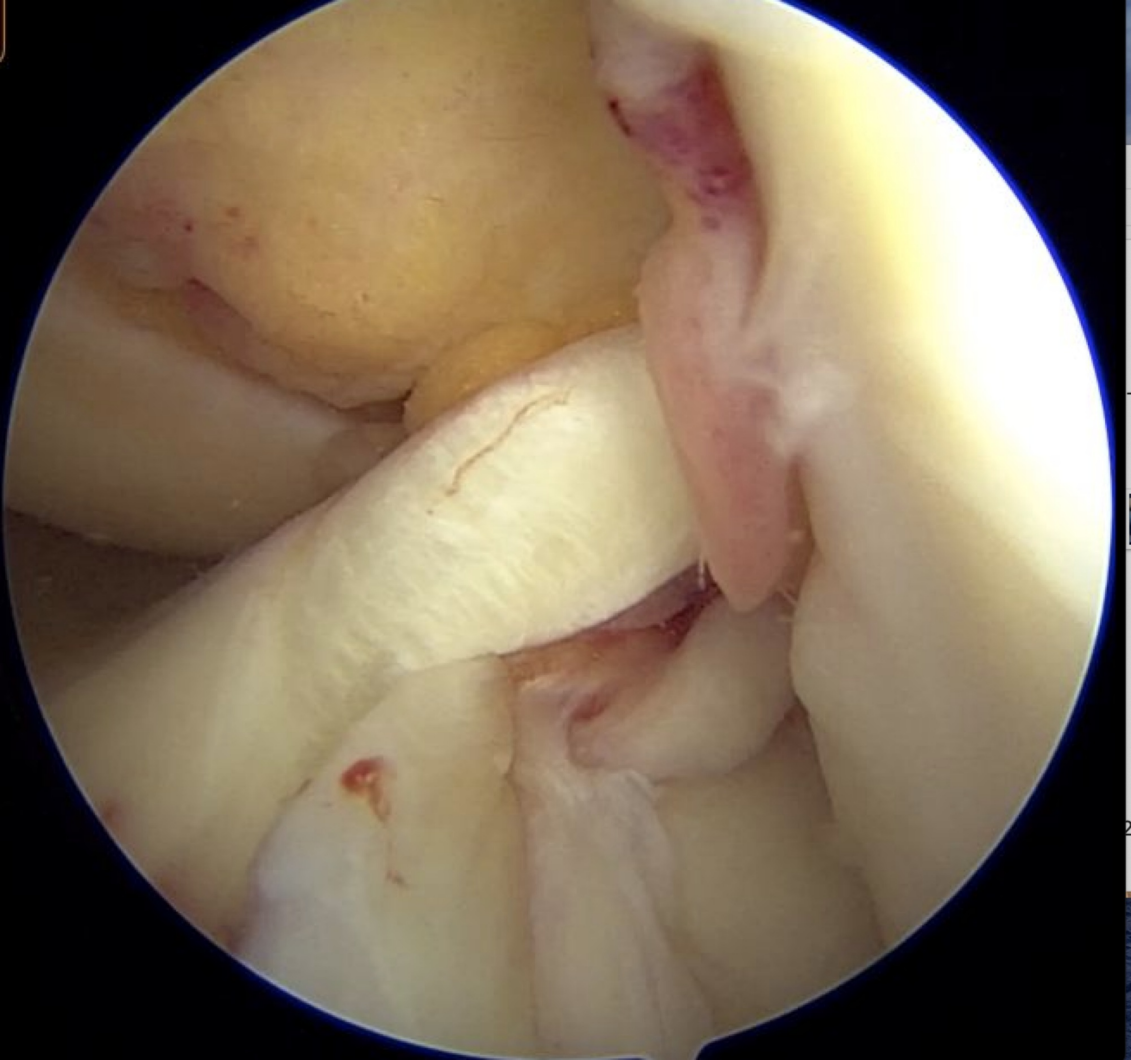
Arthroscopic view of ACL tear visualized through anterolateral portal. ACL, anterior cruciate ligament

Semitendinosus (ST) graft and gracilis graft were harvested from ipsilateral leg. Gracilis graft was prepared to reconstruct MPFL. If the harvested ST graft is of inadequate dimensions for ACL reconstruction, then contralateral hamstring was used. Through standard anterolateral and anteromedial portals, ACL tear was visualized (Figure 3). Femoral and tibial tunnels for ACL reconstruction were made.

**Figure 4:**
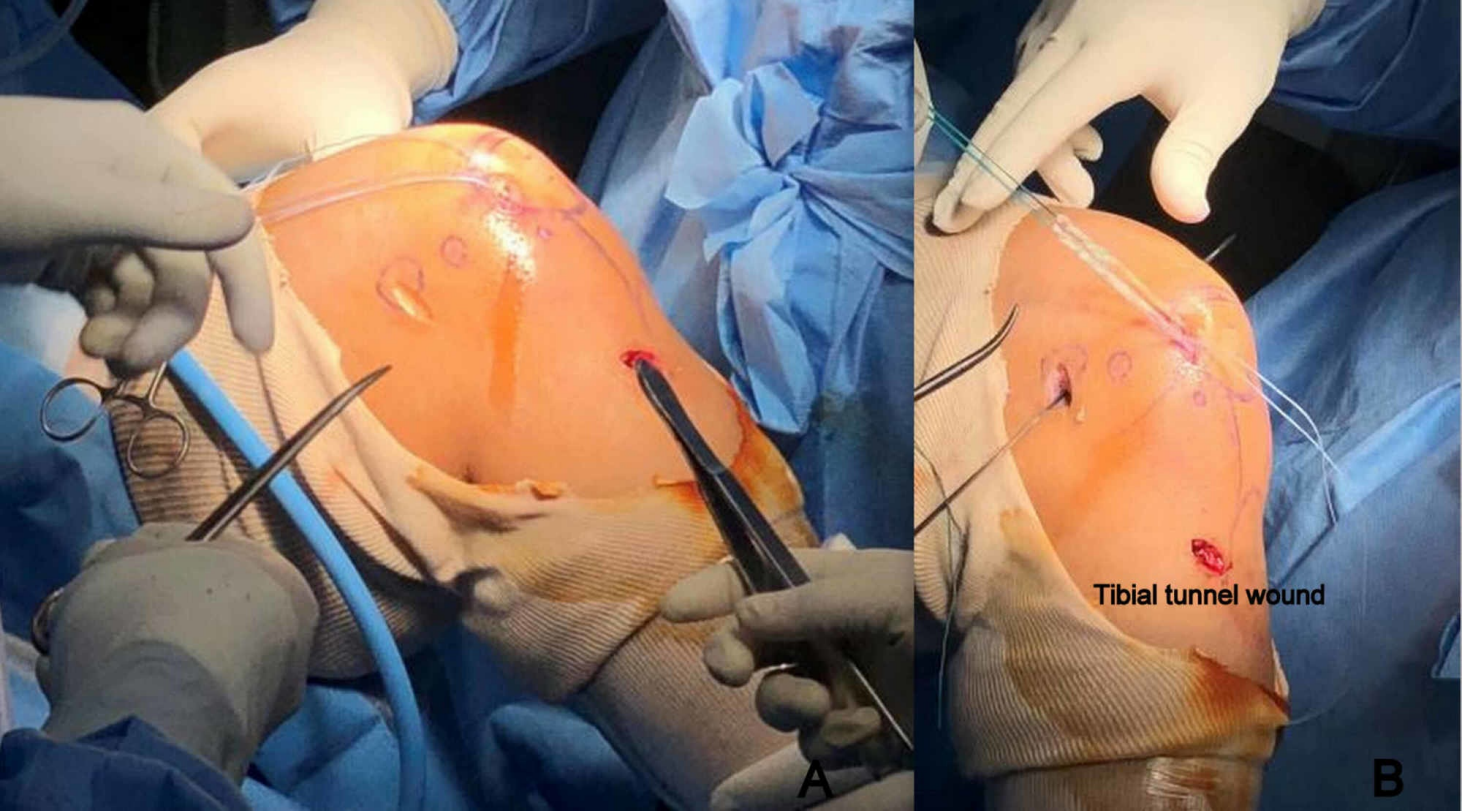
Clinical image showing A) transosseus suture loop secured to patella and B) patellar fixation of graft with sutures to medial border of patella.

**Figure 5:**
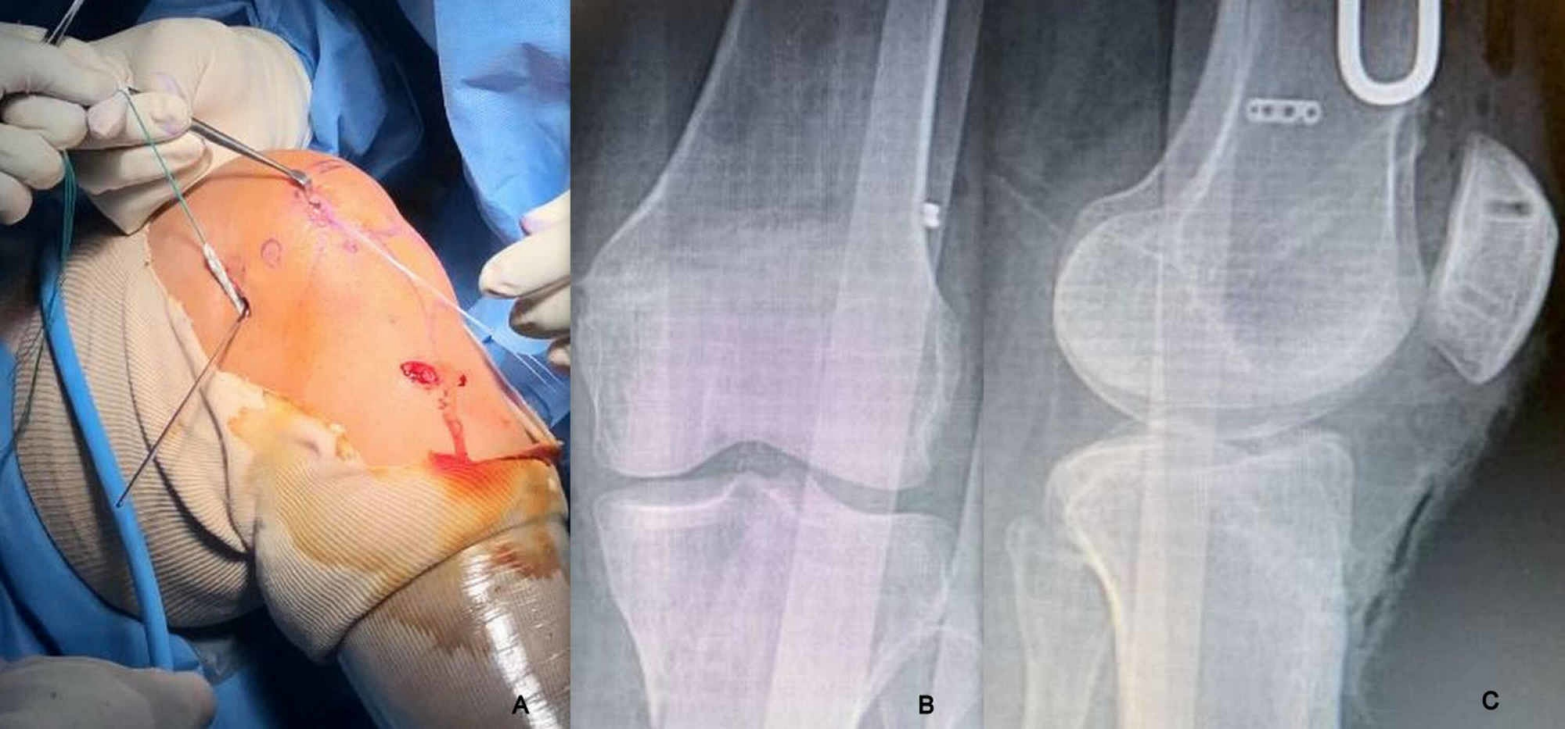
A) Clinical image of gracilis graft being tunneled under layer 2 and secured to femur isometric point; B) Immediate postoperative AP radiograph; C) Immediate lateral radiographs showing proper tunnel positions. AP, anteroposterior

For MPFL reconstruction, patellar fixation of graft was done using two tunnel transosseus suture loop technique with the graft secured medially over the patella without going through patellar tunnels (Figure 4A,B). The graft was then passed between layer 2 and layer 3 of medial aspect of knee and isometric fixation to the femur was done using an interference screw (Figure 5A). Patella was found to be centrally tracking after reconstruction. Following MPFL fixation, prepared ST graft was passed through femoral and tibial tunnels and fixed using suspensory button proximally and interference screw distally to reconstruct ACL. Postoperative radiographs were taken to confirm proper tunnel placement (Figure 5B,C). Patients were made to partial weight bear for the first three weeks.

Follow-up

Rehabilitation protocol was different from isolated ACL reconstruction in that ROM was started after week 1. Patient 1 and patient 2 had restricted knee ROM postoperatively. However, with continued rehabilitation, ROM improved and the patients regained full range at one-year follow-up. Both have returned to preinjury activity level at one-year follow-up. They had no episodes of instability at five years follow-up with satisfactory Kujala and IKDC knee scores.

In view of knee stiffness in the initial three weeks with patients 1 and 2, we started immediate aggressive ROM rehab for patient 3. She did not experience any difficulty and regained full range at 2 months follow-up.

## Discussion

Simultaneous occurrence of ACL and patellofemoral instability is rare with limited literature available on management [5-6]. To the best of our knowledge, only two studies are available on combined reconstruction of ACL and MPFL [5-6]. With regard to mechanism of injury, all our patients sustained valgus and external rotation force to their affected leg, which is the most common reason for both these tears [8]. 

In the present series, two patients had MRI evidence of ACL/MPFL tear and one patient had isolated ACL tear without MPFL rupture. We went ahead with ACL and MPFL reconstruction in all three patients. Though MRI is the gold standard for identifying MPFL tears, decision for surgery should be based on both clinical examination and imaging findings. Though one patient had normal MRI, as there was evidence of lateral patellar tracking on CT and clinical suspicion of patellar instability, both ligaments were addressed. The role of conservative treatment is limited only to those patients with negative patellar apprehension and partial MPFL tear on imaging.

Macura and Veselko had described the use of quadriceps tendon for reconstructing both these ligaments. They used deeper portion of quadriceps tendon for MPFL, while superficial portion was utilized for ACL reconstruction [5]. Hiemstra et al. had described ACL reconstruction along with concomitant MPFL imbrication in five patients and reconstruction in 13 patients (8-allografts, 7-hamstring autografts) [6]. We utilized ST grafts for single bundle ACL reconstruction and gracilis graft for MPFL reconstruction.

With respect to sequence of reconstruction, grafts are harvested first to minimize surgical time for graft preparation. ACL femoral and tibial tunnels are drilled and focus is shifted towards reconstructing MPFL. Initially the prepared gracilis graft is fixed to the patella, then tunneled under layer 2 and fixed to the isometric point on the femur using interference screw. The ST graft is then passed through ACL tunnels and secured.

Sillanpaa et al. in their series of six concurrent ACL/MPFL injuries stated the requirement of second surgery following ACL reconstruction in two cases due to continuing episodes of instability [8]. Hiemstra et al. in their series performed patellar stabilization along with revision of ACL reconstruction in three cases [6]. This suggests the importance of MPFL in preventing ACL reconstruction failures. Though the second patient in our series did not have radiographic evidence of MPFL tear, there was a strong clinical suspicion in view of positive patellar apprehension and history of patellar dislocation.

Hiemstra et al. reported good outcomes in 17/20 patients with knee stiffness in two patients and cyclops lesion in one case, which resolved with physiotherapy and arthroscopy respectively [6]. Macura and Veselko also got good results in both their patients with return to normal activities [5]. In the present report, 2/3 patients experienced knee stiffness in the initial three weeks. In view of this, we started to implement accelerated rehab from day one postsurgery.

## Conclusions

Concurrent ACL and MPFL tear is a rare injury combination that is usually missed. Clinical examination along with imaging with MRI and CT is essential in patients with ACL tear and patellar instability. Simultaneous reconstruction of both these ligaments in a single sitting offers good results.

Patellar instability should not be neglected in the setting of anteroposterior tibiofemoral instability. Identifying and treating MPFL tear is a must to avoid instability post ACL reconstruction. Failure to address this can be one of the reasons for ACL graft failure.
